# Mapping Urban Extent Using Luojia 1-01 Nighttime Light Imagery

**DOI:** 10.3390/s18113665

**Published:** 2018-10-29

**Authors:** Xi Li, Lixian Zhao, Deren Li, Huimin Xu

**Affiliations:** 1 State Key Laboratory of Information Engineering in Surveying, Mapping and Remote Sensing, Wuhan University, Wuhan 430079, China; lixi@whu.edu.cn (X.L.); drli@whu.edu.cn (D.L.); 2 Collaborative Innovation Centre of Geospatial Technology, Wuhan 430079, China; 3 School of Economics, Wuhan Donghu University, Wuhan 430212, China; 4 Key Laboratory of the Ministry of Land and Resources for Law Evaluation Engineering, Wuhan 430074, China

**Keywords:** nighttime light imagery, LJ1-01 data, urban areas, human settlement index, VIIRS DNB

## Abstract

Luojia 1-01 satellite, launched on 2 June 2018, provides a new data source of nighttime light at 130 m resolution and shows potential for mapping urban extent. In this paper, using Luojia 1-01 and VIIRS nighttime light imagery, we compared several methods for extracting urban areas, including Human Settlement Index (HSI), Simple Thresholding Segmentation (STS) and SVM supervised classification. According to the accuracy assessment, the HSI method using LJ1-01 data had the best performance in urban extent extraction, which presented the largest Kappa Coefficient value, 0.834, among all the results. For the urban areas extracted by VIIRS based HSI method, the largest Kappa Coefficient value was 0.772. In contrast, the largest Kappa Coefficient values obtained by STS method were 0.79 and 0.7512 respectively when using LJ1-01 and VIIRS data, while for SVM method the values were 0.7829 and 0.7486 when using Landsat-LJ and Landsat-VIIRS composite data respectively. The experimented results demonstrated that the utilization of nighttime light imagery can largely improve the accuracy of urban extent extraction and LJ1-01 data, with a higher resolution and more abundant spatial information, can lead to better identification results than its predecessors.

## 1. Introduction

Over the past half-century, most regions of the world, especially developing countries, have experienced a phase of high-speed urbanization [1,2,3]. In China, since the implementation of the reform and opening-up policy, the urbanization rate has increased by about 1% per year, from 17.9% in 1978 to 58.5% in 2017 [4]. The rapid development of urbanization has brought dramatic changes in urban areas, as well as a series of regional socio-economic and ecological problems caused by disorderly urban sprawl [5,6,7,8,9,10,11]. Therefore, accurate and timely measurement of urban areas, which is vital for analyzing urbanization dynamics and controlling problems mentioned above, has become an important topic in urban research [12,13,14,15,16].

As an objective and real-time data source, remote sensing satellite images have been widely used in urban area mapping and urbanization monitoring. Currently, coarse resolution images, such as the nighttime light (NTL) data have proven effective in urban extent extraction at regional and global scales [17,18,19,20,21]. At cloud-free nights, the visible light images of the earth’s surface detected by remote sensors are remotely sensed NTL images. Unlike daytime remote sensing images, the city lights recorded by the NTL images are closely related to human activities [22]. Therefore, it provides a unique perspective for socio-economic analysis and has been widely used in the fields of regional economy [23,24,25,26,27], urbanization [28,29,30], power consumption estimation [31], conflict assessment [32,33,34] and so forth. According to previous research, the Defense Meteorological Satellite Program’s Operational Linescan System (DMSP/OLS) data and the Day/Night Band (DNB) in the Visible Infrared Imaging Radiometer Suite (VIIRS) on the Suomi National Polar-orbiting Partnership Satellite are the two most commonly used NTL data sources [35,36]. Launched in the 1970s, the DMSP/OLS has a rich archive of data but it also has some shortcomings such as coarse spatial resolution (~2.7 km), blooming effects, saturation in urban cores and lack of on-board calibration, which may lead to misestimates of urban areas [31,37,38,39]. While the VIIRS DNB data, as a successor to the DMSP/OLS, has provided significant improvements such as finer spatial resolution (~740 m), on-board calibration and wider radiometric measurement range, largely reducing the saturation and blooming problems of the DMSP/OLS data [40,41,42,43].

Generally, the urban extent extraction methods using NTL data can be divided into three types, that is, thresholding-based, classification-based and index-based methods [44,45,46]. The thresholding-based method selects suitable values (local or global) to segment images [45,47,48]. As single-threshold has been proven to be problematic for urban areas at different levels of development, a multiple-threshold method is widely adopted in urban extent extraction. Shi et al. [49] utilized statistical data from the government as reference and when the urban areas extracted results and the reference data produced the minimum difference, the DNB values used for extraction were determined as the optimal thresholds. Zhou et al. [50] developed a cluster-based method to map urban areas, where a logistic regression model was used for potential urban clusters and optimal thresholds were estimated by cluster size and overall NTL magnitude. The classification-based method is to recognize NTL data as a kind of grayscale image and apply it to various classification algorithms. Jing et al. [51] utilized the integration of NTL and MODIS data and evaluated the accuracy of four classification algorithms for urban extent extraction. Xu et al. [52] and He et al. [53] mapped urban areas in China using the integrated NTL, normalized difference vegetation index (NDVI) and land surface temperature (LST) support vector machine (SVM) classification method. Nevertheless, the accuracy of classification methods is largely influenced by the selection of training samples, while solid a priori knowledge is not always available in the majority of cases. Besides, this process is labor-intensive and time-consuming and the accuracy of extraction results may suffer from the design of class schemes [54,55]. In the way of index-based methods, Lu et al. [56] proposed the human settlement index (HSI) by integrating DMSP/OLS NTL and MODIS NDVI data to extract urban areas in southeastern China. Zhang et al. [57] developed a vegetation adjusted NTL urban index (VANUI) to study urban structures on the global scale. Liu et al. [58] proposed a normalized urban area composite index (NUACI), involving the normalized difference water index (NDWI) and enhanced vegetation index (EVI) to increase the accuracy of urban extent extraction. However, limited by the spatial resolution of data sources, these methods are applied at regional or global scales in most cases.

In June 2018, Luojia 1-01 (LJ1-01), a new generation of NTL remote sensing satellite, was launched in China successfully. It has provided dramatic improvements over its predecessors, in terms of increased spatial resolution (~130 m), high radiometric quantization (14 bits) with a swath of 250 km. This new generation of NTL data source brings new possibilities and insights to the study of urban extent extraction, especially the improvement of image spatial resolution, which may not only increase the accuracy of urban extent mapping but also show more details of urban structure. Furthermore, urban extent extraction using LJ1-01 NTL data can to some extent fill the gaps in the field of NTL remote sensing in local-scale research.

The main purpose of this paper was to assess the accuracy of urban extent extraction at the city scale using LJ1-01 nighttime light imagery. For comparison, index-based, thresholding-based and classification-based methods were all adopted in the experiment. Besides, VIIRS DNB imagery were also utilized to reveal the difference between two kinds of NTL data. Finally, the Google Map high-resolution satellite images were used as reference and a visual interpretation method was applied for accuracy assessment.

## 2. Study Area and Data

### 2.1. Study Area

Wuhan, situated in the central part of China, is the capital city of Hubei Province. Wuhan covers an area of 8494.41 km^2^ and had a population of 10.89 million by the end of 2017 [59]. Due to the confluence of the Yangtze River and its greatest branch—the Han River, Wuhan is divided into three districts, namely Wuchang, Hankou and Hanyang. Wuhan has been known as the “City of Hundred Lakes”, where urban water area accounts for 1/4 of the total area. It has also been regarded as the “Thoroughfare to Nine Provinces”, serving as the transportation junction which links the east and west, as well as the north and the south [60]. Since the implementation of the reform and opening-up policy in 1978, the whole Chinese society has undergone dramatic socio-economic development, which accommodates urban expansion. Wuhan has also experienced an impressive social and economic transformation, such as industrialization and rapid urbanization and has become one of the fastest-growing cities in China during the past three decades.

### 2.2. Data

#### 2.2.1. LJ1-01

LJ1-01, launched on 2 June 2018, is the first satellite in the Luojia-1 scientific experimental satellite series owned by Wuhan University. It is the first remote sensing satellite focusing on nighttime light in China and also the first low-orbit satellite with earth observation and satellite navigation enhancement functions. LJ1-01 is a 20 kg-level micro-nano satellite, equipped with a high sensitivity night-light camera that has a spectral bandwidth of 0.319 μm. It can obtain high-precision nighttime light imagery with a dynamic range of up to 14 bits at night, with a spatial resolution of 130 meters and a swath of 250 km. It will provide an objective basis for the research of socio-economic parameter estimation, eco-environmental disaster monitoring, major event assessment, public health and other fields, carrying out dynamic monitoring of macroeconomic operations in China and the world. The data is free to download at the High-Resolution Earth Observation System of the Hubei Data and Application Center (http://59.175.109.173:8888/, accessed in June 2018). Since the radiometric calibration for LJ1-01 imagery is still under improvement, we used digital number (DN) value in this experiment for analysis. The comparison of parameters for DMSP/OLS, NPP/VIIRS and LJ1-01 NTL data are shown in Table 1.

#### 2.2.2. VIIRS DNB

VIIRS DNB Cloud-Free Composites (version 1) are a suite of average radiance composite images, where the data impacted by stray light, lightning, lunar illumination and cloud-cover have been excluded. The products are produced in 15 arc-second geographic grids, spanning the globe from 75 N latitude to 65 S. In this research, the VIIRS DNB data was utilized for comparison with LJ1-01 NTL data in urban extent extraction. Data were downloaded from the NOAA National Geophysical Data Center (https://ngdc.noaa.gov/eog/viirs/download_dnb_composites.html). Since the monthly composite images of June 2018 have not been released yet, the images of May 2018 were selected for the experiment.

#### 2.2.3. Landsat 8 OLI

Landsat 8 Operational Land Imager (OLI) Level-2 data for the study area were obtained from the United States Geological Survey (USGS) website (https://earthexplorer.usgs.gov/). Level-2 data products, also called surface reflectance products, provide an estimate of the surface spectral reflectance at a 30-m spatial resolution in the absence of atmospheric scattering or absorption [61]. Since the Landsat image in June 2018 was partly covered by clouds, the closest cloud-free image in April 2018 was selected for this research. The data was utilized in both HSI and SVM methods to compare their performance in urban extent extraction.

#### 2.2.4. Other Auxiliary Data

**Figure 1:**
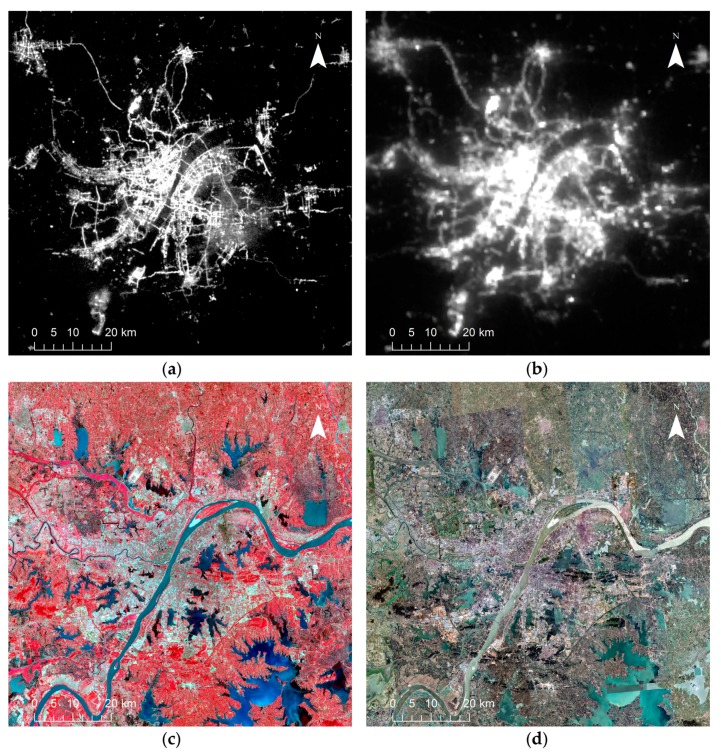
Study area under different data sources: (**a**) LJ1-01 NTL image in June 2018; (**b**) VIIRS DNB image in May 2018; (**c**) Landsat 8 OLI false color composite (R/G/B = 5/4/3) in April 2018; (**d**) Google Map remote sensing satellite image in 2018.

Google Map remote sensing satellite images, with a spatial resolution of 1 m, were utilized as reference data in the visual interpretation accuracy assessment. The study area under different data sources are shown in Figure 1.

## 3. Methods

### 3.1. Data Preprocessing

First of all, the data in the study area—Wuhan city, was clipped from the original datasets. Then, for consistency among different data sources, the LJ1-01 NTL, the VIIRS DNB, the Landsat 8 OLI products and the Google Map satellite images were all re-projected to Albers Conical Equal Area projection. After that, a bilinear interpolation algorithm was applied to resample the LJ1-01 NTL and VIIRS DNB images to the same resolution as that of the Landsat data (i.e., 30 m) and each image has a size of 3000 × 3000 pixels. In addition, since land-reflected moon light has not been excluded from the monthly composite products of VIIRS DNB data, it is necessary to remove background noise before use. In this research, all pixel values in VIIRS imagery were subtracted by 0.5 first, then pixels with negative DN values were assigned values of zero.

To apply the HSI method developed by Lu et al. [56], the NDVI image was generated first, as Equation (1) shows. However, this method has been proven to be difficult to separate urban areas and water bodies accurately [62]. Thus, to enhance the performance of the HSI, the NDWI image was also generated as a mask to remove water areas from the original HSI result. The NDWI proposed by Mcfeeters [63] was derived as Equation (2):

(1) $$\;NDVI=\frac{NIR-R}{NIR+R}\;$$

(2) $$\;NDWI=\frac{G-NIR}{G+NIR}\;$$

where NIR, R and G are Landsat 8 OLI surface reflectance images in near-infrared, red and green band respectively.

### 3.2. Urban Extent Extraction Based on Different Methods

#### 3.2.1. Human Settlement Index

The Human Settlement Index, proposed by Lu et al. [56], is a method of extracting residential sites from the integration of nighttime light data and the NDVI index. In theory, NDVI images generated by Equation (1) have values ranging from −1 to 1. However, as the LJ1-01 NTL data has a dynamic radiometric range of 14 bits, the DN values in the study area ranging from 0 to 1879. Thus, it is necessary to match the NTL data with the range of NDVI index.

First, pixels with zero values were masked out and a natural logarithmic transformation was adopted to the LJ1-01 NTL images to maintain the rich details in the nighttime light. Then, the data was normalized as Equation (3):

(3) $$\;NTL_{nor}=\frac{NTL-NTL_{min}}{NTL_{max}-NTL_{min}}\;$$

where $NTL_{nor}$ is the normalized value of the LJ1-01 NTL image, $NTL_{max}$ and $NTL_{min}$ are the minimum and maximum values in the LJ1-01 NTL image respectively. Then the HSI was generated as Equation (4):

(4) $$\;HSI=\frac{\left(1-NDVI\right)+NTL_{nor}}{\left(1-NTL_{nor}\right)+NDVI+NTL_{nor}\times NDVI}\;$$

After that, regions with DN values greater than 0 in the NDWI image were regarded as water bodies, the mask was generated and the corresponding regions in the HSI image were removed. Lastly, multiple thresholds (from 0.25 to 1.2 with an interval of 0.05) were applied to extract urban areas from the HSI image.

The processing of VIIRS DNB data was exactly the same as that of LJ1-01 data mentioned above. The logarithm transformation and normalization algorithm were adopted first, then the HSI was generated, with water bodies removed by NDWI. Lastly, multiple thresholds were applied to extract urban areas from the HSI image.

#### 3.2.2. Simple Thresholding Segmentation

In order to compare with the result of the HSI method in Section 3.2.1, the Simple Thresholding Segmentation (STS) method was also adopted in this research using LJ1-01 NTL and VIIRS DNB data respectively [64]. Since it was difficult to determine the optimal threshold for urban extent extraction, multiple thresholds have been tested and finally, 1 to 20 with an interval of 1 have been selected as threshold values to extracted urban areas from the two kinds of NTL imagery.

#### 3.2.3. SVM Supervised Classification

As mentioned in the Introduction section, thresholding-based, classification-based and index-based methods are the three most commonly used methods for urban extent mapping. Thus, in this research, the SVM supervised classification method was also adopted for comparison [65,66]. First, the normalized difference built-up index (NDBI) was generated. The NDBI index was proposed by Zha et al. [67] for urban built-up areas extraction using Landsat TM data. When applied to Landsat 8 OLI data, it was derived as [68]:

(5) $$\;NDBI=\frac{SWIR1-NIR}{SWIR1+NIR}\;$$

where SWIR1 and NIR are Landsat 8 OLI surface reflectance images in shortwave infrared 1 and near-infrared band respectively. Then, in addition to Landsat band 1~7, the generated NDBI image was also added as an additional band to enhance the information of built-up areas in Landsat data. The image was classified into five land-cover types presented in the study area, that is, bare soil, water bodies, vegetation, built-up land in high reflectance and built-up land in low reflectance. For each land-cover type, over 1000 pixels were selected as training samples based on interpretation of high-resolution images from Google Earth and separability for all the type-pairs were greater than 1.8. After performing the SVM classification, bare soil, water bodies and vegetation in the results were merged into non-urban areas, while the built-up land covers were recognized as urban areas.

In order to compare the results using different datasets, NTL data was also utilized in this experiment for classification-based urban extent extraction. The resampled LJ1-01 and VIIRS nighttime light imagery was added to Landsat band 1~7 as an additional band respectively and the Landsat-LJ and Landsat-VIIRS composite data were generated. Then the SVM classifier was applied using the same training samples as above. Lastly, the extraction results went for accuracy assessment, which will be presented in Section 3.3.

### 3.3. Accuracy Assessment

A visual interpretation method was utilized to quantify the performance of different urban extent extraction methods presented in Section 3.2. Google Map remote sensing satellite imagery was recognized as reference data, that is, “ground truth” data, with a spatial resolution of 1 m.

First, 800 randomly sampled points in the study area were generated. Then each point was labeled as “urban area” or “non-urban area” by visual interpretation using Google Map satellite images. After that, extracted urban areas obtained in Section 3.2 were recognized as “labeling results” and the confusion matrix was established for each method to perform the accuracy assessment.

The confusion matrix is a specific table that is often used to describe the accuracy of classification results [69]. Generally, rows in the confusion matrix correspond to classes, while columns correspond to predicted classes (or vice versa). Several metrics are often used as performance assessment measures based on confusion matrix, that is, Producer’s Accuracy (PA), User’s Accuracy (UA), Overall Accuracy (OA) and Kappa Coefficient (KC). PA measures the omission error, indicating the probability that an urban pixel is correctly identified. UA measures the commission error, indicating the probability that a labeled urban pixel is truly urban. OA indicates the proportion of pixels that are correctly identified and lastly, KC indicates the agreement between “labeling results” and “ground truth” by combining both types of errors, which can provide a more comprehensive assessment [70,71]. These metrics are computed as:

(6) $$\;PA_{k}=\frac{x_{kk}}{x_{k+}}\;$$

(7) $$\;UA_{k}=\frac{x_{kk}}{x_{+k}}\;$$

(8) $$\;OA=\frac{\sum^{}x_{kk}}{N}\;$$

(9) $$\;KC=\frac{N\sum^{}x_{kk}-\sum^{}x_{k+}x_{+k}}{N^{2}-\sum^{}x_{k+}x_{+k}}\;$$

where $x_{kk}$ is the number of pixels that are correctly identified, $x_{k+}$ is the total number of pixels that belong to class k, $x_{+k}$ is the total number of pixels identified as class k and N is the total number of pixels in the dataset.

## 4. Results

**Figure 2:**
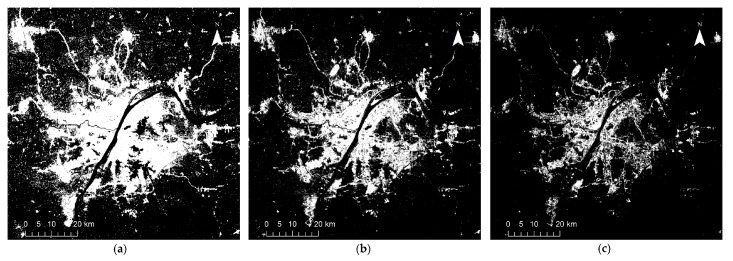
Extracted urban areas of Wuhan by HSI method using LJ1-01 NTL data: (**a**) threshold = 0.4; (**b**) threshold = 0.8; (**c**) threshold = 1.2.

**Figure 3:**
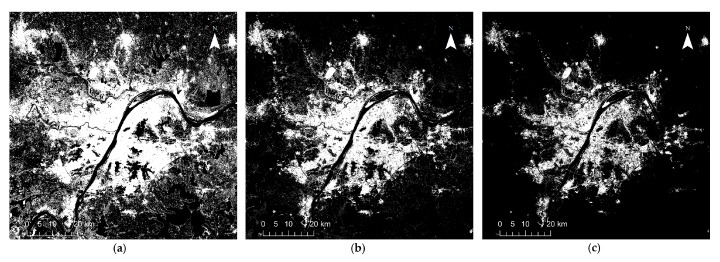
Extracted urban areas of Wuhan by HSI method using VIIRS DNB data: (**a**) threshold = 0.6; (**b**) threshold = 0.9; (**c**) threshold = 1.2.

**Figure 4:**
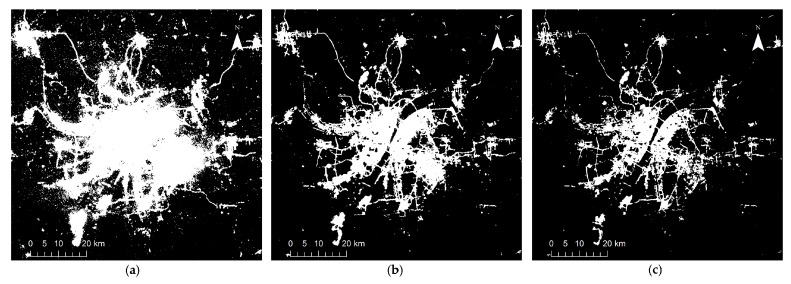
Extracted urban areas of Wuhan by STS method using LJ1-01 NTL data: (**a**) threshold = 2; (**b**) threshold = 10; (**c**) threshold = 18.

**Figure 5:**
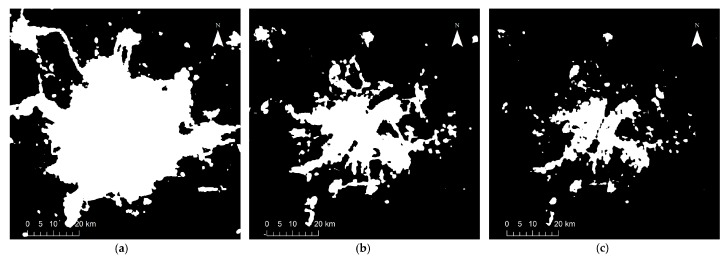
Extracted urban areas of Wuhan by STS method using VIIRS DNB data: (**a**) threshold = 2; (**b**) threshold = 10; (**c**) threshold = 18.

**Figure 6:**
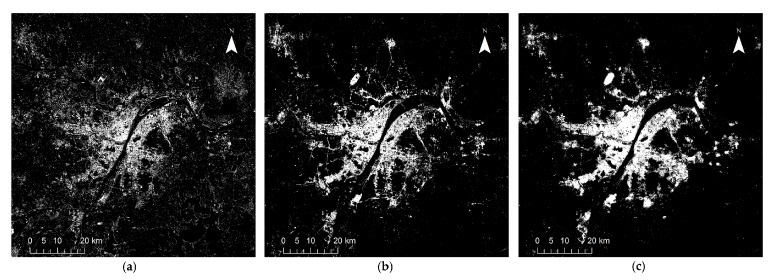
Extracted urban areas of Wuhan by SVM method using: (**a**) Landsat data only; (**b**) Landsat and LJ1-01 composite data; (**c**) Landsat and VIIRS composite data.

Figure 2, Figure 3, Figure 4, Figure 5 and Figure 6 show some of the urban extent extraction results based on different methods. Figure 2 and Figure 3 show urban areas of Wuhan extracted by the HSI method using LJ1-01 and VIIRS data respectively, while Figure 4 and Figure 5 show urban areas extracted by STS method using LJ1-01 and VIIRS data respectively. And urban areas shown in Figure 6 were extracted by SVM method using Landsat only, Landsat-LJ composite and Landsat-VIIRS composite data. By comparing different results, it can be found that urban extent extracted by the HSI method using LJ1-01 and VIIRS data were generally similar. In the results derived from the STS method, a lot of spatial information within the city was lost, especially when using VIIRS data. While in contrast, the urban extent extracted by SVM method were overestimated in suburban areas especially when using Landsat data only.

**Figure 7:**
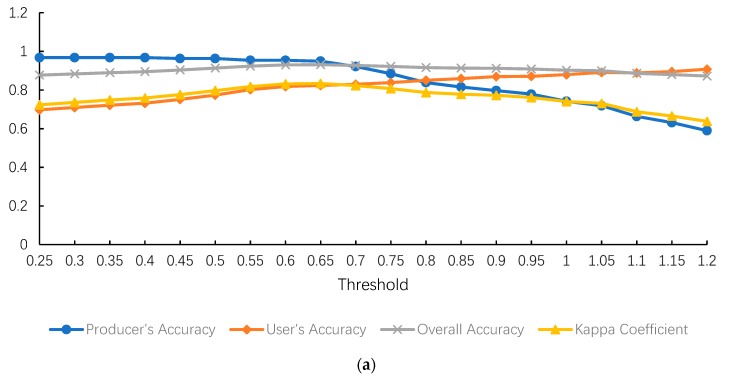

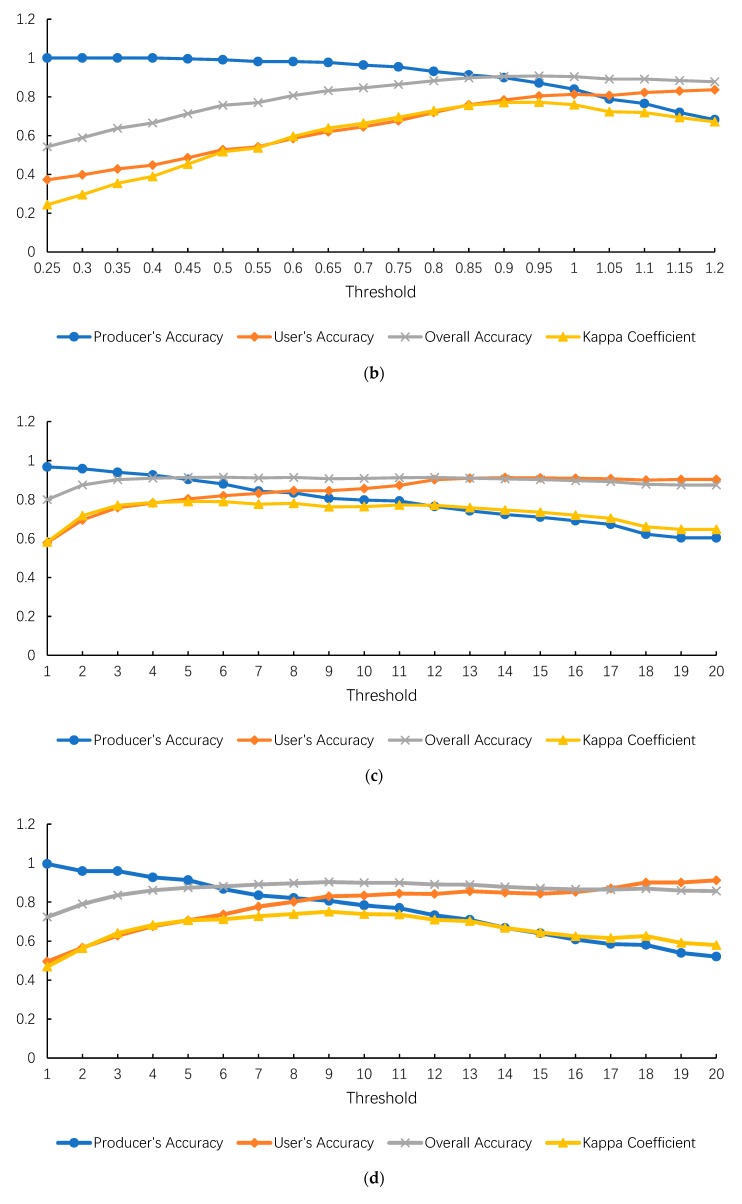

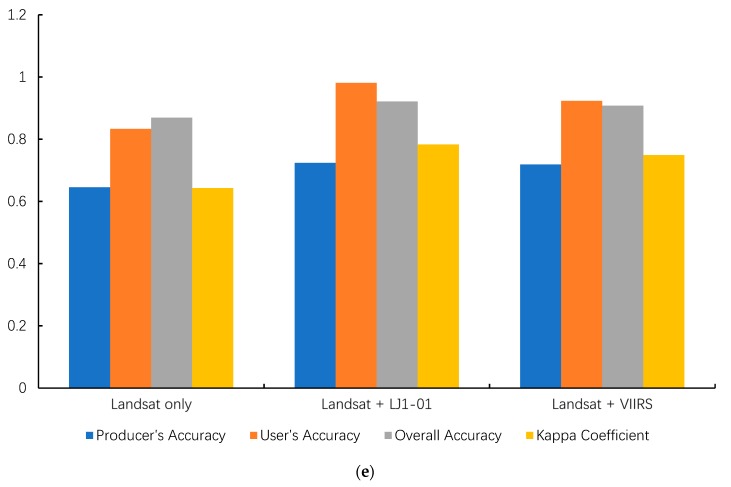
Accuracy assessment of urban extent extraction for: (**a**) HSI method using LJ1-01 data; (**b**) HSI method using VIIRS data; (**c**) STS method using LJ1-01 data; (**d**) STS method using VIIRS data; (**e**) SVM method.

The accuracy assessment for the performance of different methods are presented in Figure 7. It can be found that the urban extent extracted by the HSI method using LJ1-01 data with a threshold of 0.65 showed the largest KC value, 0.834, among all the results. Compared with other methods, the HSI method using LJ1-01 data had the best performance, with all KC values larger than 0.797 when the thresholds were between 0.5 and 0.75. In terms of the HSI method using VIIRS data, the KC values increased gradually from 0.2434 with the increase of the thresholds, reached the maximum value of 0.772 when the threshold was 0.95 and then began to slowly decline. In contrast, the KC value obtained by STS method showed different change trends from HSI method. When using LJ1-01 data, the KC values remained at a relatively higher level around 0.77 when the thresholds were between 3 and 12, then began to decrease with the increase of the thresholds. When using VIIRS data, the change trend was similar but the maximum KC value only reached 0.7512, which was lower than using LJ1-01 data. As for the SVM method, the KC values for using Landsat only, Landsat-LJ composite and Landsat-VIIRS composite data were 0.6427, 0.7829 and 0.7486 respectively.

In terms of PA, the HSI method using LJ1-01 and VIIRS data both maintained significantly high PA values when the thresholds were less than 0.7 and they also had similar change trends. In contrast, the PA values of the STS method using LJ1-01 and VIIRS data were relatively low and dropped sharply with increasing thresholds. In terms of UA, all the methods that used LJ1-01 data kept at a relatively high level and the SVM method using Landsat-LJ composite data had the best result. In the matter of OA, LJ1-01 data still had better performance than VIIRS data. The largest OA values for HSI, STS and SVM method when using LJ1-01 data were 0.9313, 0.915 and 0.9213 respectively, all larger than those of VIIRS data used ones.

## 5. Discussion

### 5.1. The Advantages of NTL Data in Urban Area Extraction

In this research, we compared several commonly used methods for extracting urban areas, including NTL based and non-NTL based methods. The experimental results demonstrated that the adding of NTL to Landsat data can improve the accuracy of urban extent extraction results.

Urban areas shown in Figure 6a were extracted by the SVM method using Landsat data only, while urban areas shown in Figure 6b,c were extracted by the same method, except that the data were replaced with Landsat-LJ composite and Landsat-VIIRS composite data respectively. As can be seen from the results, with the integration of NTL data, the overestimation in urban suburbs has been largely reduced and the number of small patches has also decreased. According to Figure 7, after adding VIIRS and LJ1-01 data, the KC values of SVM method increased from 0.6427 to 0.7486 and 0.7829 respectively, while the values of PA, UA and OA metrics all increased in varying degrees.

**Figure 8:**
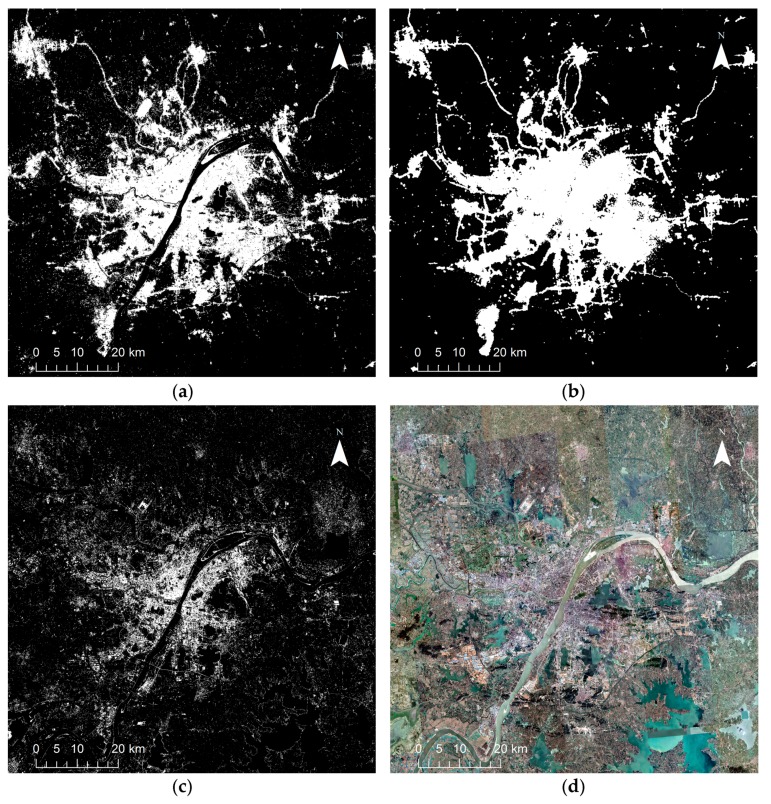
Urban extent extraction results with the largest Kappa Coefficient for: (**a**) HSI method (LJ1-01 data used and threshold = 0.65); (**b**) STS method (LJ1-01 data used and threshold = 5); (**c**) SVM method using Landsat data only; (**d**) Google Map satellite image for comparison.

For a more intuitive comparison of NTL based and non-NTL based methods, urban extent extraction results with the largest KC values for HSI and STS method are presented in Figure 8a,b respectively, while Figure 8c presents the result of SVM method using Landsat data only, along with the Google Map satellite image in Figure 8d for comparison. It can be clearly seen that the HSI method (LJ1-01 data used, threshold = 0.65) had the best extraction result of urban extent, while the SVM method using only Landsat data had the worst. Specifically, the STS method (LJ1-01 data used, threshold = 5) shows a large overestimation in urban cores, while SVM method contrarily shows an underestimation in urban cores and overestimation in urban suburbs, resulting in a large number of redundant small patches. This is consistent with the accuracy assessment results in Figure 7. The values of OA and KC metrics both exhibited the same decreasing trends among these three results, that is, HIS > STS > SVM. This finding demonstrates that the SVM method using only Landsat data to extract urban extent may not be accurate enough. It is difficult to separate bare soils from urban land, which may cause overestimation in urban suburbs. The utilization of NTL data, which is able to represent luminous built-up areas at night, can greatly improve the accuracy of urban extent extraction.

### 5.2. The Advantages of LJ1-01 Data Compared with VIIRS

**Figure 9:**
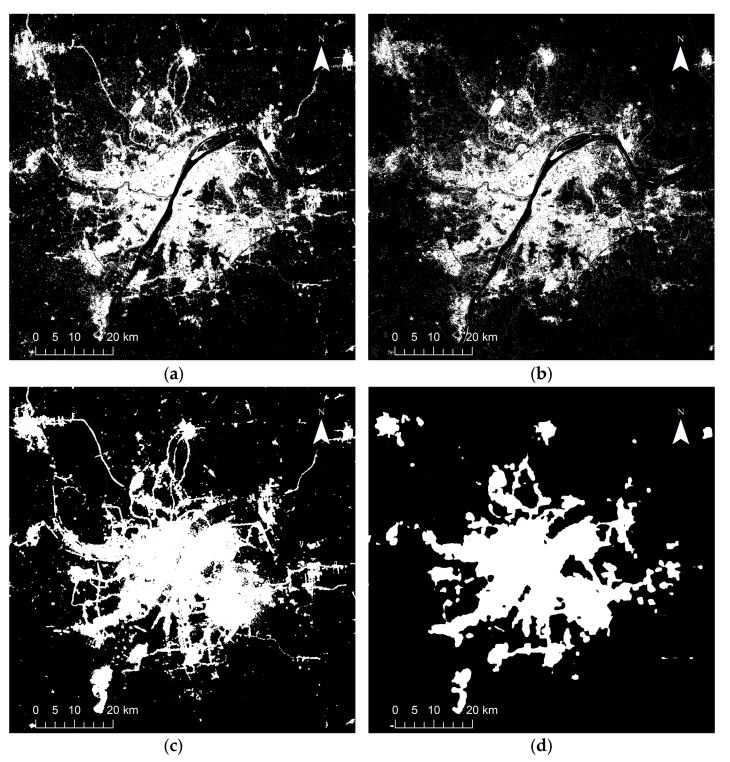
Urban extent extraction results with the largest Kappa Coefficient for: (**a**) HSI method using LJ1-01 data (threshold = 0.65); (**b**) HSI method using VIIRS data (threshold = 0.95); (**c**) STS method using LJ1-01 data (threshold = 5); (**d**) STS method using VIIRS data (threshold = 9).

In this research, we also compared the performance of different NTL data in urban extent extraction. LJ1-01 NTL and VIIRS DNB images were utilized to contrast two main nighttime light data sources. Figure 9a,b displays the urban extent extraction results with the largest KC values for the HSI method using LJ1-01 and VIIRS data respectively and the results with the largest KC values for STS method are shown in Figure 9c,d. It can be seen that HSI methods using both LJ1-01 and VIIRS data were effective in urban extent extraction. In addition to human settlements, the luminous bridges and roads at night can also be identified accurately. However, the urban areas extracted based on VIIRS data were more fragmented with a large number of small patches and many urban pixels have not been identified. In contrast, the LJ1-01 based HSI method exhibited better performance, which extracted urban extent more accurately with less fragments and patches. This result is supported by the accuracy assessment according to Figure 7. The largest KC value for VIIRS based HSI method was 0.772, while it increased to 0.834 for the LJ1-01 based method. In terms of other metrics, the PA value increased from 0.871 to 0.9493, the UA value from 0.8043 to 0.824 and the OA value from 0.9075 to 0.9313 for VIIRS and LJ1-01 data. Moreover, it can be seen from Figure 9c,d that when applying the STS method, the extracted urban extent based on LJ1-01 data contained more urban spatial information than the VIIRS data. This finding can be obtained from the accuracy assessment in Figure 7 as well. The largest KC value for LJ1-01 based STS method, 0.79, was higher than that for the VIIRS based method. In terms of the SVM method, the PA, UA, OA and KC values using Landsat-LJ composite data were 0.7235, 0.9813, 0.9213 and 0.7829 respectively, all of which were larger than the corresponding values using Landsat-VIIRS composite data. The above results demonstrate that the LJ1-01 NTL data, which has a higher spatial resolution, can bring more abundant spatial details in urban extent extraction and lead to better identification results compared with VIIRS DNB data.

### 5.3. Prospects for the Future

According to the experiments and analysis above, we have concluded that LJ1-01 NTL data, with a spatial resolution of 130 m, contains more abundant spatial information of urban heterogeneity and can lead to a more accurate result when used in urban extent extraction. However, there are still some limitations in the application of LJ1-01 NTL data, which is worth more efforts in the future.

As a new nighttime light imagery source, the Luojia 1-01 satellite was launched in June 2018, which means that only images from June 2018 to now can be obtained for the time being. The lack of multi-temporal images makes LJ1-01 NTL data temporarily unable to be applied to long-term urban dynamic monitoring. However, if LJ1-01 NTL data can be integrated with other nighttime light data which has similar spatial resolution and long-time archived images, such as the International Space Station (ISS) imagery, this problem can be alleviated to a certain extent. The ISS imagery is a kind of multispectral photograph taken by astronauts in space using digital cameras, which has both daytime and nighttime archives [72]. All the photographs can be freely downloaded at “Gateway to Astronaut Photography of Earth” (https://eol.jsc.nasa.gov/), which is run by the National Aeronautics and Space Administration (NASA). Compared with other commonly used nighttime light data, the ISS imagery has several advantages, such as higher spatial resolution, three spectral bands in the visible range and variable overpass times [73]. It has an abundant data archive since last century and during the past decade, the quality of its imagery, especially nighttime light imagery has increased dramatically. However, for most global cities, its high-quality imagery archiving is not enough to do research on urban dynamic monitoring. With the release of LJ1-01 data, the integration of LJ1-01 and ISS nighttime light images may provide a solution to this problem. By integrating various types of high-resolution NTL data for a consistent observation, we can identify urban expansion areas between different periods in a fine scale, as well as better understand the dynamic patterns of urban land expansion. Moreover, further analysis in other geographical regions or at a global scale is to be performed. By combining high-resolution NTL data from different sources and other remote sensing data, an accurate extraction of urban extent in different periods and regions can be achieved. Therefore, a continuing mapping of urban dynamics at the global scale will also be possible in the future.

## 6. Conclusions

In this research, we compared different methods of urban extent extraction in Wuhan city, including a Human Settlement Index using both LJ1-01 and VIIRS images, Simple Thresholding Segmentation using both LJ1-01 and VIIRS images and SVM supervised classification using Landsat and NTL composite data. The performance of different methods was assessed by reference samples from visual interpretation of Google Map satellite images. The results demonstrate that the HSI method using LJ1-01 NTL data had the best performance in urban extent extraction, which can not only accurately extract human settlements but also identify luminous bridges and roads at night. In addition, it presented the largest Kappa Coefficient value, 0.834, among all the results when the threshold was 0.65, while other metrics remained at a high level. The relevant findings of this research are as follows:

(1) Compared with non-NTL methods, the addition of NTL data can largely improve the accuracy of the extraction results. For SVM classification method, the values of all accuracy assessment metrics have increased in varying degrees after adding LJ1-01 and VIIRS data respectively. By comparing the urban extent extraction results which have the largest KC values for different methods, we can find out that non-NTL based SVM classification method is likely to cause overestimation in urban suburbs, while NTL based methods exhibited better performance according to the accuracy assessment.

(2) LJ1-01 NTL data, which contains more spatial details, can lead to better identification results than VIIRS DNB data in urban extent extraction. The urban areas extracted by the VIIRS based HSI method were fragmented with a large number of small patches, while the LJ1-01 based method identified urban land more accurately. The same finding can be obtained from the accuracy assessment. The largest KC value for the VIIRS based method was 0.772, while it increased to 0.834 for the LJ1-01 based method. As for the STS and SVM methods, the LJ1-01 NTL data also presented better performance compared with VIIRS DNB data.

(3) LJ1-01, as a new generation of nighttime light imagery, has provided higher spatial resolution, wider radiometric measurement range and richer urban dynamic information over its predecessors. It displays a great potential for urban extent extraction at city scale and is bound to be more widely used in urban mapping. However, the current lack of multi-temporal images temporarily limits its application in urban dynamic monitoring. By integrating LJ1-01 with other high-resolution nighttime light imagery, we may find a solution to this problem. Moreover, the integration of high-resolution nighttime light imagery and other remote sensing data may provide a possibility to map urban extent in different periods and regions and then monitor urban dynamics continuously at the global scale in the future.

## Figures and Tables

**Table 1:** Comparison of parameters for different NTL data.

Parameters	DMSP/OLS	NPP/VIIRS	LJ1-01
Available Period	1992–2013	November 2011–present	June 2018–present
Country	The U.S.	The U.S.	China
Spatial Resolution	2.7 km	740 m	130 m
Swath	3000 km	3000 km	250 km
Spectrum Range	0.5–0.9 μm	0.5–0.9 μm	0.46–0.98 μm
Radiometric Resolution	6 bits	14 bits	14 bits
Saturation	Saturated	Not saturated	Not saturated
